# The HIV protease inhibitor darunavir prevents kidney injury via HIV-independent mechanisms

**DOI:** 10.1038/s41598-019-52278-3

**Published:** 2019-11-01

**Authors:** Xiaobo Gao, Alan Rosales, Heidi Karttunen, Geetha M. Bommana, Buadi Tandoh, Zhengzi Yi, Zainab Habib, Vivette D’Agati, Weijia Zhang, Michael J. Ross

**Affiliations:** 1 Division of Nephrology, Albert Einstein College of Medicine/Montefiore Medical Center, Bronx, NY USA; 2 BronxCare Health System, Bronx, NY USA; 3 Division of Nephrology, Icahn School of Medicine at Mount Sinai, New York, NY USA; 4 New York University, New York, NY USA; 5 Department of Pathology, Columbia University, College of Physicians & Surgeons, New York, NY USA; 6 Department of Development and Molecular Biology, Albert Einstein College of Medicine, Bronx, NY USA

**Keywords:** Molecular medicine, Nephrology

## Abstract

HIV-associated nephropathy (HIVAN) is a rapidly progressive kidney disease that is caused by HIV infection of renal epithelial cells with subsequent expression of viral genes, including *vpr*. Antiretroviral therapy ameliorates HIVAN without eradicating HIV from the kidneys and the mechanism by which it protects kidneys is poorly understood. Since HIV protease inhibitors have “off target” cellular effects, we studied whether darunavir, the most commonly prescribed protease inhibitor, protects kidneys from HIV-induced injury via mechanisms independent of HIV protease and viral replication. Renal epithelial cells were transduced with lentiviruses encoding HIV (lacking protease and reverse transcriptase), Vpr, or vector control. Darunavir attenuated HIV and Vpr-induced activation of Stat3, Src, Erk, and cytokines, which are critical for HIVAN pathogenesis. We then studied HIV-transgenic mice, which develop HIVAN in the absence of HIV protease or reverse transcriptase. Mice were treated with darunavir, zidovudine, darunavir + zidovudine, or control. Darunavir and darunavir + zidovudine reduced albuminuria and histologic kidney injury and normalized expression of dysregulated proteins. RNA-seq analyses demonstrated that darunavir suppressed HIV-induced upregulation of immune response genes in human kidney cells. These data demonstrate that darunavir protects against HIV-induced renal injury via mechanisms that are independent of inhibition of HIV protease.

## Introduction

The widespread use of antiretroviral therapy (ART) has led to a dramatic decrease in AIDS-related mortality^1^. As persons living with HIV (PLWH) live longer, non-AIDS conditions such as chronic kidney disease (CKD) have become increasingly important causes of patient morbidity and mortality. Kidney disease is the fourth-leading disease contributing to death in PLWH in the United States^2,3^. HIV-associated nephropathy (HIVAN) is an important cause of end stage renal disease (ESRD) in PLWH, usually occurs in late in the course of HIV-infection^4^, and predominantly strikes persons of African Ancestry^5^. Since approximately 25.7 million of the 37 million HIV-positive persons worldwide live in Africa, 43% of whom do not receive ART^6^, HIV-related kidney disease is a particularly important problem in Africa^7^.

HIVAN was the most rapidly rising cause of ESRD in African Americans aged 25–64 in the early 1990s^8^. Though some reports suggested that zidovudine (AZT) monotherapy reduced progression of HIVAN^9^, it was not until the widespread adoption of combination ART regimens that included reverse transcriptase (RT) and protease inhibitors (PI), that the incidence of ESRD due to HIVAN suddenly plateaued^10^.

ART initiation is associated with improved kidney function in patients with advanced HIV/AIDS with CKD of unknown etiology^11,12^, suggesting that ART may have efficacy in treating kidney diseases other than HIVAN. Since some ART agents have been associated with increased risk of CKD progression^13^, it is critical to better understand the effects of ART upon the kidney in the context of HIVAN and other forms of CKD. Importantly, a recent study suggested that the PI saquinavir is effective in treating HIV-*negative* patients with steroid-dependent nephrotic syndrome^14^, suggesting that PI may protect the kidneys via mechanisms independent of suppression of HIV replication.

While it is widely accepted that ART has efficacy in the prevention and treatment of HIVAN, the mechanism by which ART protects the kidneys is uncertain. In a patient with HIVAN who underwent kidney biopsy before and after clinical and histologic resolution of disease after ART treatment, there was no decrease in HIV RNA in renal parenchymal cells after treatment^15^, suggesting ART does not prevent/treat HIVAN by reducing the burden of HIV infection of renal epithelial cells.

Tg26 is the most commonly used animal model of HIVAN and these mice are transgenic for a g*ag/pol*-deleted HIV provirus under control of the endogenous viral LTR promoter^16^. Since *gag* encodes major viral structural proteins and *pol* encodes reverse transcriptase (RT), protease (PR), and integrase (IN) (the targets of most ART medications), this model demonstrates that viral replication and expression of RT, PR, or IN are unnecessary for the HIVAN phenotype in mice. *In vitro* and *in vivo* models have demonstrated that expression of HIV-1 Vpr and/or Nef in renal epithelial cells, in the absence of viral replication, is a key mediator of HIVAN pathogenesis^17^. While Nef likely has important roles in glomerular injury^18–20^, Vpr induces injury in glomeruli and tubular cells^18,21,22^. Currently available ART agents do not directly target the function of Vpr or Nef.

It is rare for HIVAN to develop in ART-treated patients with undetectable viral load and CD4 count >200cells/mm^3^ ^23^. However, in two studies that reported development of HIVAN in ART-treated patients with undetectable viral load and CD4 count >200cells/mm^3^, each of the three patients was on an ART regimen that did not include a PI^24,25^. Since there has been a major shift away from use of PI-based ART regimens in PLWH toward integrase strand inhibitor (INSTI)-based regiments^26^, it is critical to determine whether PI confer particular benefit in patients with HIVAN or other forms of CKD. However, some PI have been associated with acute and chronic kidney injury due to poor water solubility leading to intratubular crystallization, and/or nephrolithiasis^27–30^. Darunavir (DRV) is now the most commonly-used PI and is associated with fewer adverse renal effects than other PIs^29–32^.

In these studies, we test our hypothesis that DRV protects the kidneys against HIV-induced kidney injury via mechanisms that are, in part, independent of HIV protease or suppression of HIV replication. We present data demonstrating that DRV prevents dysregulation of cellular pathways known to be important in the pathogenesis of HIVAN in human renal epithelial cells *in vitro* and ameliorates the HIVAN phenotype in Tg26 via HIV-PR-independent mechanisms.

## Results

### Darunavir prevents dysregulation of key cellular pathways in human tubular cells *in vitro*

**Figure 1:**
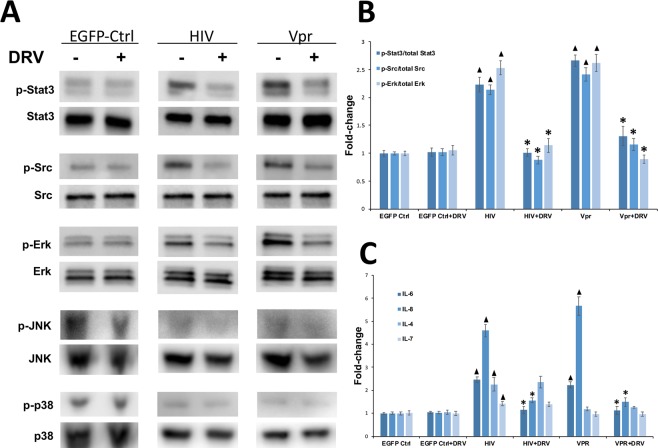
Darunavir prevented HIV and Vpr-induced dysregulation of signaling and cytokine expression in HPT1b cells. Western blotting demonstrated increased phosphorylation of Stat3, Src and Erk in cells transduced with HIVΔG/P or Vpr lentivirus compared to EGFP-control lentivirus. DRV reduced HIV- and Vpr-induced phosphorylation of Stat3, Src and Erk (**A**) and these changes were statistically significant when quantified by densitometry (**B**). Activation of JNK and p38 were not altered by HIV- or Vpr-transduction or DRV (A). qPCR analysis demonstrated that IL-6 and IL-8 expression increased after HIV and Vpr transduction but induction of IL-6 and IL-8 was suppressed by DRV (**C**). IL-4 and IL-7 expression increased after HIV but not Vpr transduction and expression was not altered by DRV (**C**) ^▲^p < 0.03 compared to EGFP-control transduced cells *p < 0.01 compared to DRV-untreated cells.

To test whether DRV can protect kidney cells from the deleterious effects of HIV via HIV protease-independent mechanisms, we studied the effects of DRV on renal tubular epithelial cells (RTEC). Conditionally immortalized human RTEC (HPT1b cells)^33,34^ were transduced with lentiviral vectors encoding either *gag/pol*-deleted HIV (based on the same provirus used in Tg26 HIVAN model, which lacks HIV protease), Vpr, or control lentivirus expressing EGFP and subsequently treated with DRV or vehicle control. Since activation of Stat3, Src, and Erk pathways are important in the pathogenesis of HIV-induced kidney injury^19^, we studied whether DRV could prevent their activation. HIV and Vpr-transduction of HPT1b cells increased phosphorylation of Stat3 (p < 0.01), Src (p < 0.01), and Erk (p < 0.01) compared to cells transduced with EGFP control lentivirus and DRV significantly prevented HIV and Vpr-induced phosphorylation of Stat3 (p < 0.01), Src (p < 0.01), and Erk (p < 0.01) (Fig. 1A,B). We also studied the effects of HIV and DRV upon activation of the mitogen activated protein kinases (MAPK) JNK and p38 in HPT1b cells. We did not detect an effect of HIV or Vpr transduction, or DRV treatment on JNK or p38 activation (Fig. 1A).

Since HIV-induced expression of proinflammatory mediators, including IL-6 and IL-8 are critical mediators of HIVAN pathogenesis^35^, we analyzed IL-6 and IL-8 expression in control, HIV- and Vpr-transfected HPT1b cells treated with or without DRV (Fig. 1C). HIV and Vpr transduction increased the expression of IL-6 (p < 0.01 and p = 0.03 respectively) and IL-8 (p < 0.01) in HPT1b cells compared to control-transduced cells. HIV and Vpr-induced upregulation of IL-6 and IL-8 was abrogated by DRV (Fig. 1C, p < 0.01). HIV also increased expression of IL-4 (p = 0.02) and IL-7 (p = 0.01) but expression was not affected by DRV. IL-4 and IL-7 expression were not changed after Vpr transduction with or without DRV (Fig. 1C).

### DRV treatment reduced glomerular and tubulointerstitial injury in Tg26 mice

**Figure 2:**
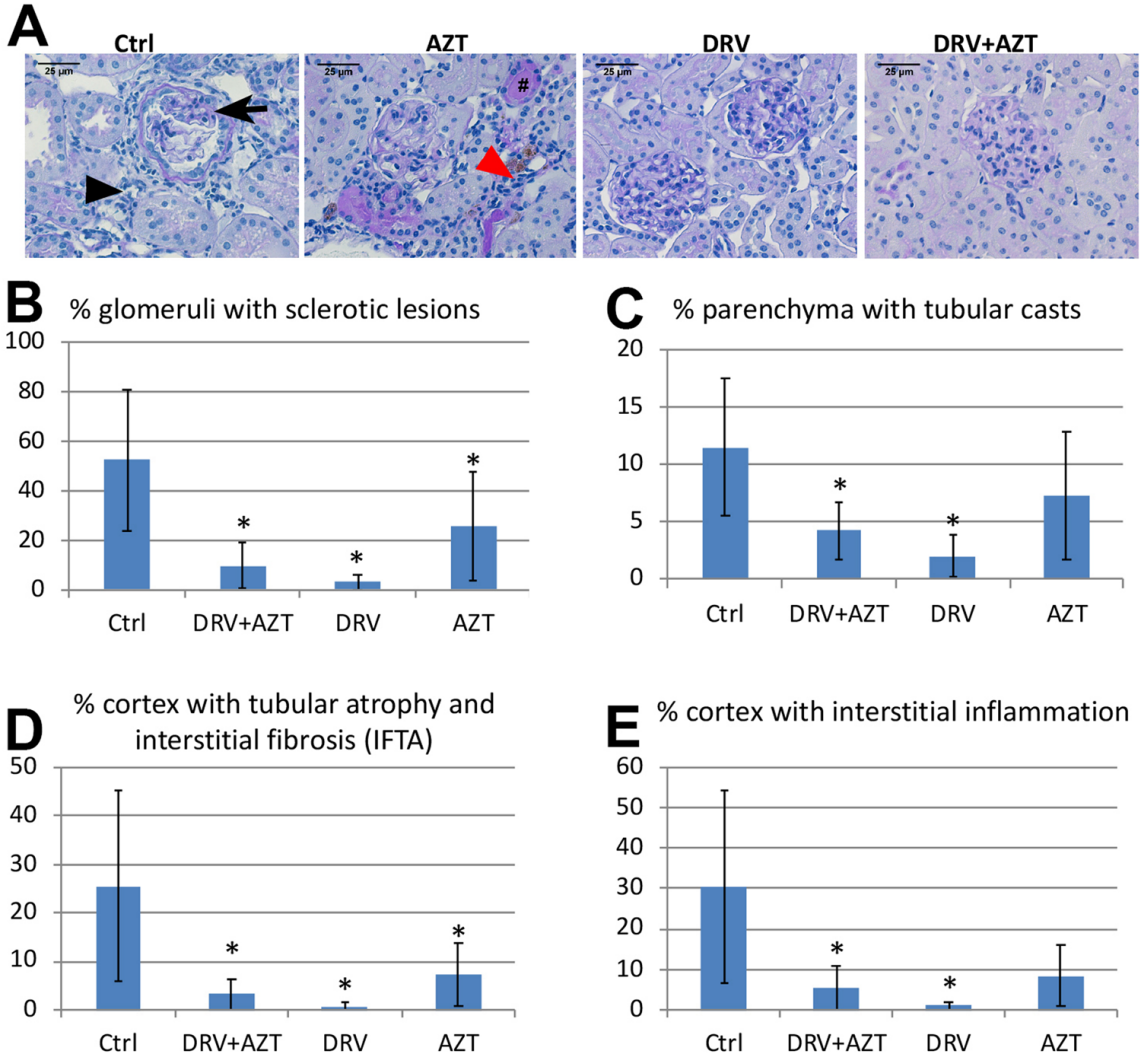
DRV reduced histologic glomerular and tubulointerstitial injury in Tg26 mice. Control-treated Tg26 kidneys had glomerulosclerosis (arrow) and tubulointerstitial injury with tubular casts (#), tubular atrophy (red arrowhead), and interstitial inflammation (black arrowhead) typical of the HIVAN phenotype. These changes were less severe in DRV- and DRV + AZT-treated Tg26 mice (**A**). Quantitative histomorphometric analyses of PAS-stained sections demonstrated that each of these indices were markedly elevated in Tg26 mice given vehicle control but DRV and DRV + AZT treated Tg26 mice had significantly reduced severity in all parameters of histologic injury compared to vehicle-treated Tg26 mice (**B**-**E**). AZT-treated mice did not have significantly improved tubular cast formation (**C**) or interstitial inflammation (**E**) but did have reduced glomerulosclerosis (**B**) and interstitial fibrosis and tubular atrophy (**D**) compared to control-treated Tg26 mice. *p < 0.05 vs control. Scale bar, 25 µm.

We next studied whether DRV can protect HIV-transgenic mice from developing the HIVAN phenotype. Since PLWH are always treated with combination ART and some studies suggested that AZT monotherapy may be protective against HIVAN^9^, we also tested the efficacy of AZT and DRV + AZT in Tg26 mice. Four-month-old heterozygous Tg26-FVB mice were assigned to 4 treatment groups and were treated daily by oral gavage for 5 weeks with DRV, AZT, DRV + AZT, or vehicle control. Control-treated Tg26 mice developed typical histopathologic findings of HIVAN, whereas glomerulosclerosis and tubulointerstitial injury were improved in Tg26 treated with DRV or DRV + AZT (Fig. 2A). Quantitative histomorphometry demonstrated that DRV and DRV + AZT groups had significantly reduced glomerulosclerosis (Fig. 2B, p = 0.015, and p = 0.029, respectively), tubular cast formation (Fig. 2C, p = 0.023 and p = 0.048, respectively), interstitial fibrosis and tubular atrophy (Fig. 2D, p = 0.025 and p = 0.017, respectively), and interstitial inflammation (Fig. 2E, p = 0.012 and p = 0.013, respectively) compared to vehicle-treated mice. AZT-treated mice did not have significant changes in tubular cast formation or interstitial inflammation but did have reduced glomerulosclerosis (Fig. 2B, p = 0.045) and interstitial fibrosis and tubular atrophy (Fig. 2D, p = 0.018).

### DRV treatment reduced albuminuria in HIV-transgenic mice

**Figure 3:**
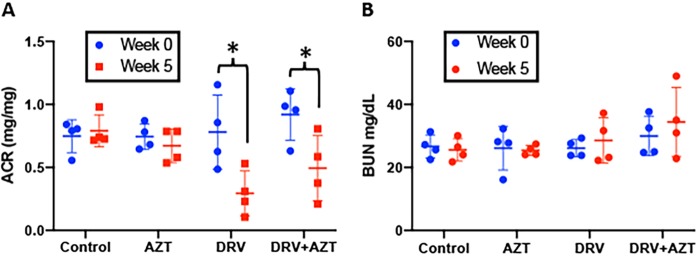
DRV treatment reduced albuminuria in HIV-transgenic mice. HIV-transgenic mice (Tg26) were treated daily for 35 days with vehicle control, AZT, DRV, or DRV + AZT. ACR did not change in mice given vehicle control (p = 0.489) or AZT (p = 0.37), but decreased in DRV (p < 0.01) and DRV + AZT groups (p = 0.04) (**A**). There was no difference in BUN between groups at baseline or after 5 weeks of treatment (**B**).

Albuminuria, which was assessed by urinary albumin/creatinine ratio (ACR), was elevated in Tg26 mice and did not change during the study period in mice treated with vehicle control (p = 0.489) or AZT (p = 0.37) (Fig. 3A). However, DRV and DRV + AZT treatment reduced albuminuria compared to control-treated Tg26 mice over 35 days of treatment (p < 0.01 and p = 0.04, respectively).

Renal function was also assessed by measurement of blood urea nitrogen (BUN). Since mice were studied at a relatively early stage of nephropathy, BUN was not grossly elevated in any group at baseline. There was no significant difference in BUN between groups after 5 weeks of treatment (Fig. 3B).

### DRV reduced molecular markers of HIV-induced injury in Tg26 mice

**Figure 4:**
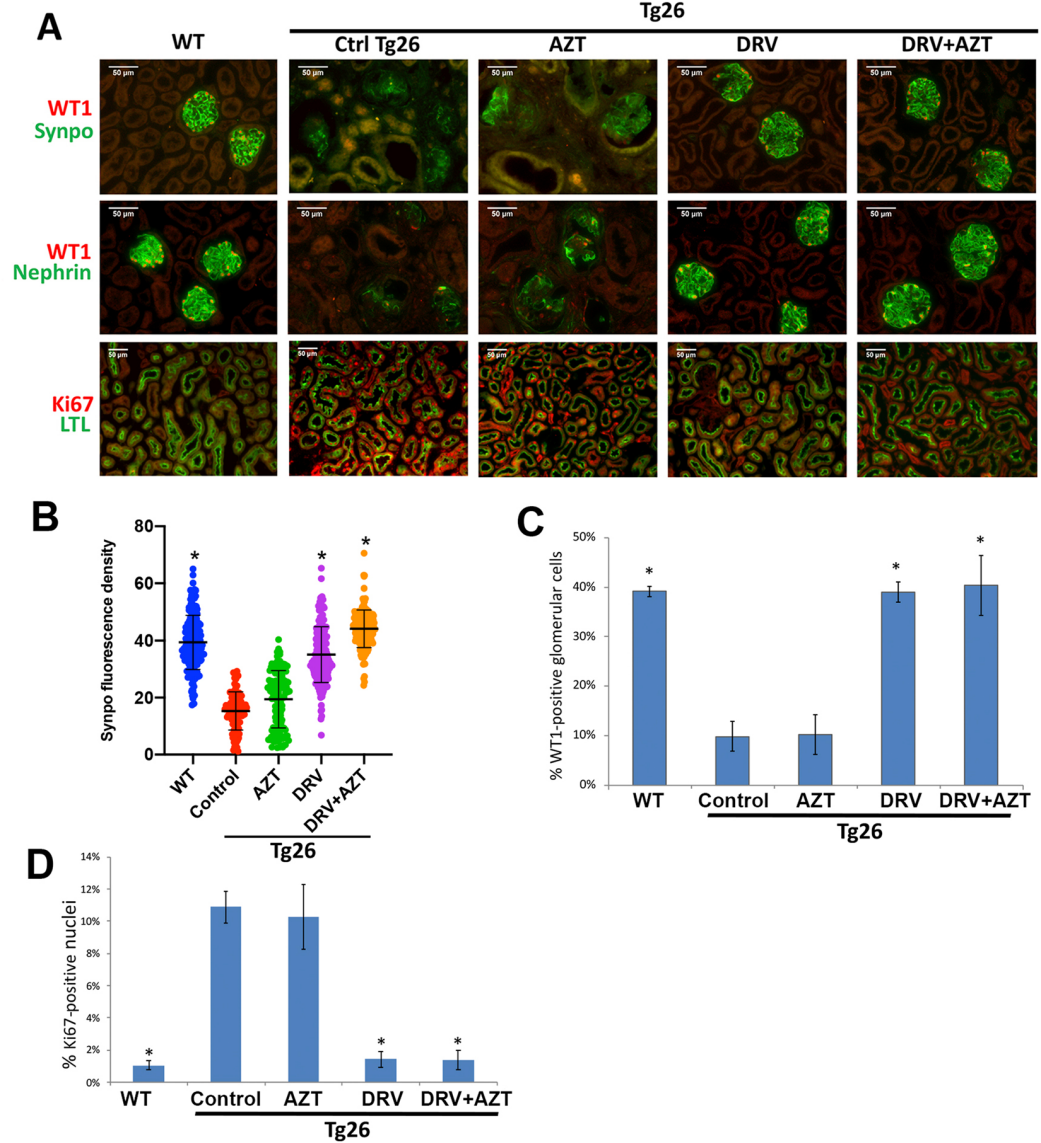
DRV reduced molecular markers of injury in Tg26 mice. Immunofluorescence microscopy demonstrated strong Synpo, Nephrin, and WT1 expression in podocytes in wild-type mice and markedly reduced expression in vehicle control- and AZT-treated Tg26 mice (**A**). Synpo, Nephrin, and WT1 expression in DRV- or DRV + AZT-treated Tg26 mice was similar to wild-type mice (**A**). Quantitative assessment of Synpo (**B**) and WT1 (**C**) demonstrated significantly higher expression in wild-type, DRV and DRV + AZT-treated Tg26 mice compared to vehicle control and AZT-treated Tg26 mice. Immunofluorescence microscopy revealed rare Ki-67-positive cells in wild-type kidneys but increased expression in Tg26 mice, particularly in proximal tubules, treated with vehicle control or AZT (**A**,**D**). The percentage of Ki-67-positive cells was reduced in DRV and DRV + AZT treated Tg26 mice (**A**,**D**) to levels similar to wild-type mice (**D**). Scale bar, 50 µm. *p < 0.01 compared to Tg26 treated with vehicle or AZT.

We then studied the effect of DRV upon expression of cellular proteins that are dysregulated in HIVAN. Synaptopodin (Synpo) and Wilms Tumor-1 (WT1) are proteins with critical roles in podocyte function whose expression is reduced in HIVAN^36,37^. Synpo and WT1 were readily detected by immunofluorescence microscopy in podocytes of wild-type mice but expression was markedly reduced in podocytes of kidneys from vehicle control- or AZT-treated Tg26 mice (Fig. 4). Synpo and WT1 expression was normalized in DRV and DRV + AZT treated Tg26 mice (Fig. 4). The podocyte-specific protein Nephrin was expressed in a pattern similar to that observed for Synpo (Fig. 4).

Ki-67 is expressed in cells in phases of the cell cycle other than G(0) and is often used as a marker of proliferating cells^38^. Wild-type kidneys had rare Ki-67-positive nuclei whereas Tg26 mice treated with vehicle control or AZT had increased Ki-67-expressing cells, most of which were proximal tubular cells, as determined by costaining with proximal tubule-specific Lotus tetragonolobus lectin (LTL) (Fig. 4A). Tg26 mice treated with DRV or DRV + AZT had a statistically significant reduction in the percentage of Ki-67-positive cells to levels similar to wild-type mice (Fig. 4D). Taken together, these results demonstrate that DRV but not AZT prevented dysregulation of several cellular proteins in Tg26 mice via mechanisms that are independent of HIV PR.

### DRV treatment prevented dysregulation of key cellular pathways in Tg26 mice

**Figure 5:**
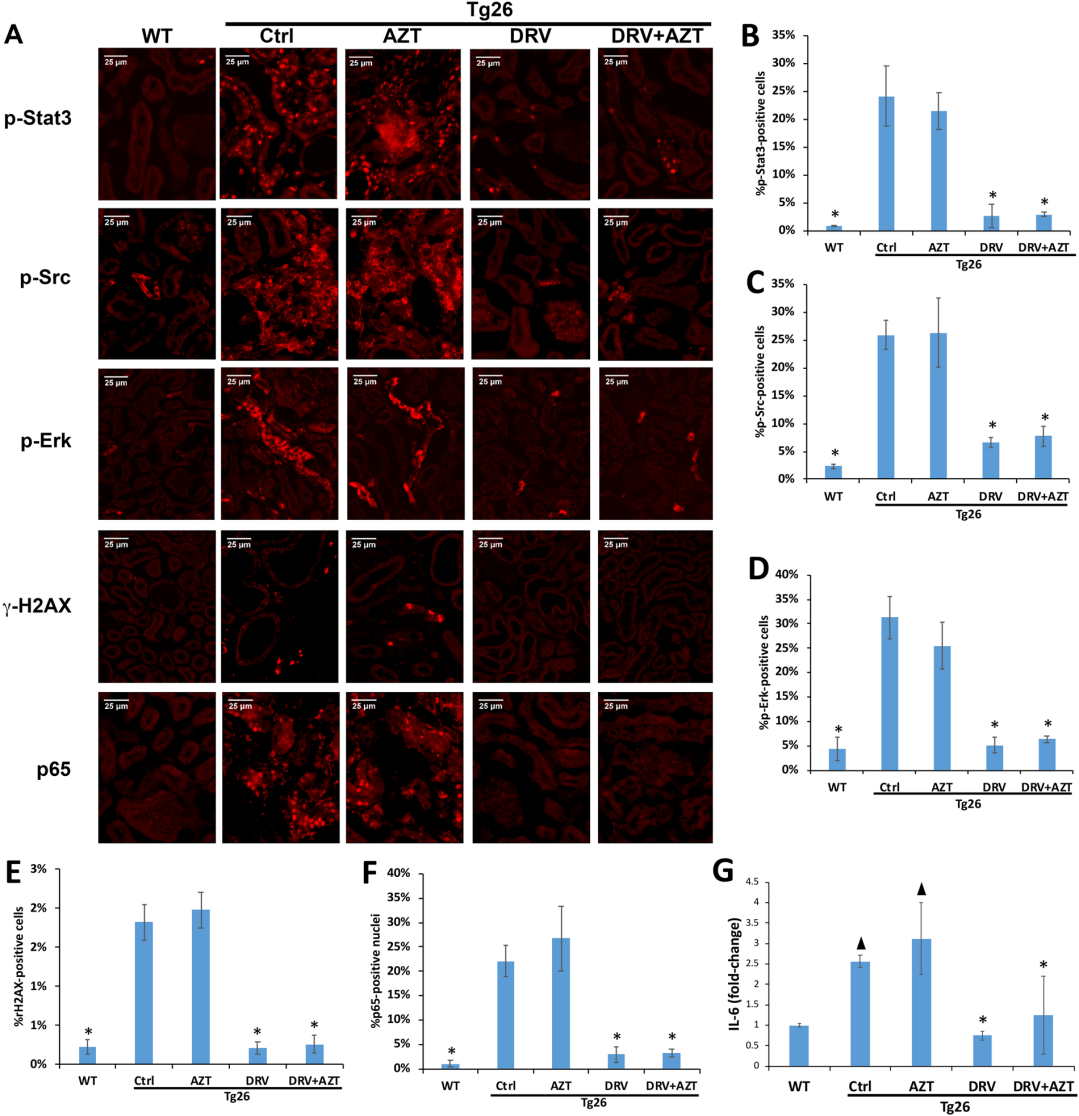
Darunavir decreased dysregulation of key cellular pathways in Tg26 kidneys. Immunofluorescence microscopy demonstrated rare cells staining for p-Stat3, p-Src, or p-Erk-positive in wild type kidneys but all were markedly increased in vehicle control and AZT-treated Tg26 mice, with much lower levels detected in DRV or DRV + AZT-treated Tg26 mice (**A**). Similarly, immunofluorescence microscopy for γH2AX and the p65 subunit of NF-kB revealed minimal nuclear staining in wild-type kidneys but higher levels in Tg26 kidneys treated with vehicle control or AZT (**A**), which was reduced in mice treated with DRV and DRV + AZT (**A**). Scale bar 25 µm, same magnification in all panels (**A**). IL-6 expression was significantly elevated in vehicle control- and AZT-treated Tg26 kidneys compared to kidneys from wild type mice (**B**). DRV and DRV + AZT treatment reduced IL-6 expression in Tg26 kidneys to levels similar to those in wild type mice (**B**). ^▲^p < 0.01 compared to wild type mice, *p < 0.01 compared to Tg26 treated with vehicle or AZT.

Immunofluorescence microscopy revealed rare p-Stat3-positive tubular epithelial cells in wild-type kidneys (Fig. 5A). However, kidneys from Tg26 mice treated with vehicle control (Fig. 5A) or AZT had markedly increased levels of p-Stat3, whereas levels were similar to wild-type in DRV or DRV + AZT treated Tg26 mice. Similarly, p-Src and p-Erk were increased in kidneys of Tg26 mice treated with vehicle control or AZT, but not in those treated with DRV or DRV + AZT (Fig. 5A).

Vpr expression induces cell cycle dysregulation and activation of the DNA damage response in RTEC, as reflected by phosphorylation of histone H2AX to form γ-H2AX^39^. Immunofluorescence microscopy revealed prominent nuclear γ-H2AX staining in RTEC in Tg26 mice treated with vehicle or AZT, but reduced γ-H2AX staining in Tg26 mice treated with DRV or DRV + AZT (Fig. 5A).

NF-κB activation also has a critical role in the pathogenesis of HIV-induced kidney injury^40–42^. Kidneys of Tg26 mice treated with vehicle or AZT, had increased nuclear p65 abundance compared to wild-type kidneys, and nuclear p65 was decreased in kidneys from DRV or DRV + AZT treated Tg26 mice (Fig. 5A), suggesting that DRV but not AZT reduced NF-κB activation in Tg26 mice.

We further studied whether DRV reduced IL-6 expression in kidneys from Tg26 mice. IL-6 was increased in vehicle control- and AZT-treated Tg26 kidneys compared to kidneys from wild-type mice (Fig. 5B). DRV and DRV + AZT treatment reduced IL-6 in Tg26 kidneys to levels similar to those in wild-type mice (Fig. 5B). Though we also found that DRV reduced IL-8 expression in HPT1b cells (Fig. 2), IL-8 could be not assessed in Tg26 mice because it is absent from the murine genome.

### Darunavir does not block 26S proteasome function

Some studies have reported that the anti-inflammatory effects of HIV protease inhibitors are mediated via blocking degradation of IkBα by the 26S proteasome, thereby preventing translocation of NF-κB heterodimers to the nucleus^14^, however, other studies reported that in PI’s do not reach sufficient concentrations in *in vivo* to inhibit the 26S proteasome^43^. We therefore tested the ability of darunavir to block the 26S proteasomal trypsin-like, chymotrypsin-like, and caspase-like proteolytic activity in HPT1b cells. Cells were incubated with fluorogenic substrates to monitor each proteasomal peptidase activity. We did not detect significant effects of darunavir upon any of these proteasomal functions (Figure S1).

### Cellular pathways affected by darunavir treatment

**Figure 6:**
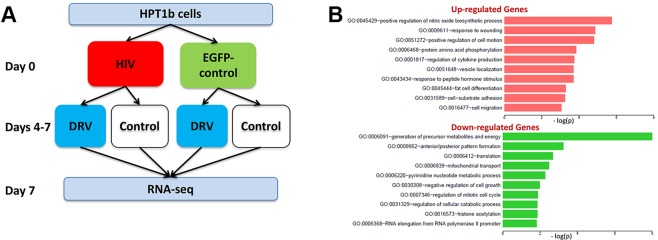
RNA-seq analysis of effects of HIV and DRV upon gene expression in HPT1b cells. Schematic plan for RNA-seq studies to evaluate effects of DRV upon gene expression in HPT1b cells (**A**). Gene ontology biological functions of differentially expressed genes at day 7 after transduction with HIV compared to EGFP control (**B**).

The cellular molecular target(s) of DRV that mediate its protective in the kidney are unknown. We therefore performed RNAseq studies to identify upstream cellular pathways that mediate the effects of DRV (experimental schema in Fig. 6A). We first identified genes that were differentially expressed in HPT1b cells 7 days after transduction with HIV compared to EGFP control virus and vehicle-treated controls. 1,015 genes were upregulated in HIV-transduced cells and gene ontology pathway analysis revealed that the cellular pathways most affected by the upregulated genes were those involved in nitric oxide synthesis, injury and cytokine responses, cell motility, and protein phosphorylation (Fig. 6B). Pathway analysis of the 895 downregulated genes in HIV-transduced cells revealed that the most significantly downregulated cellular pathways were those involved in energy metabolism/mitochondrial function, anterior/posterior patterning, and protein translation (Fig. 6B).

**Figure 7:**
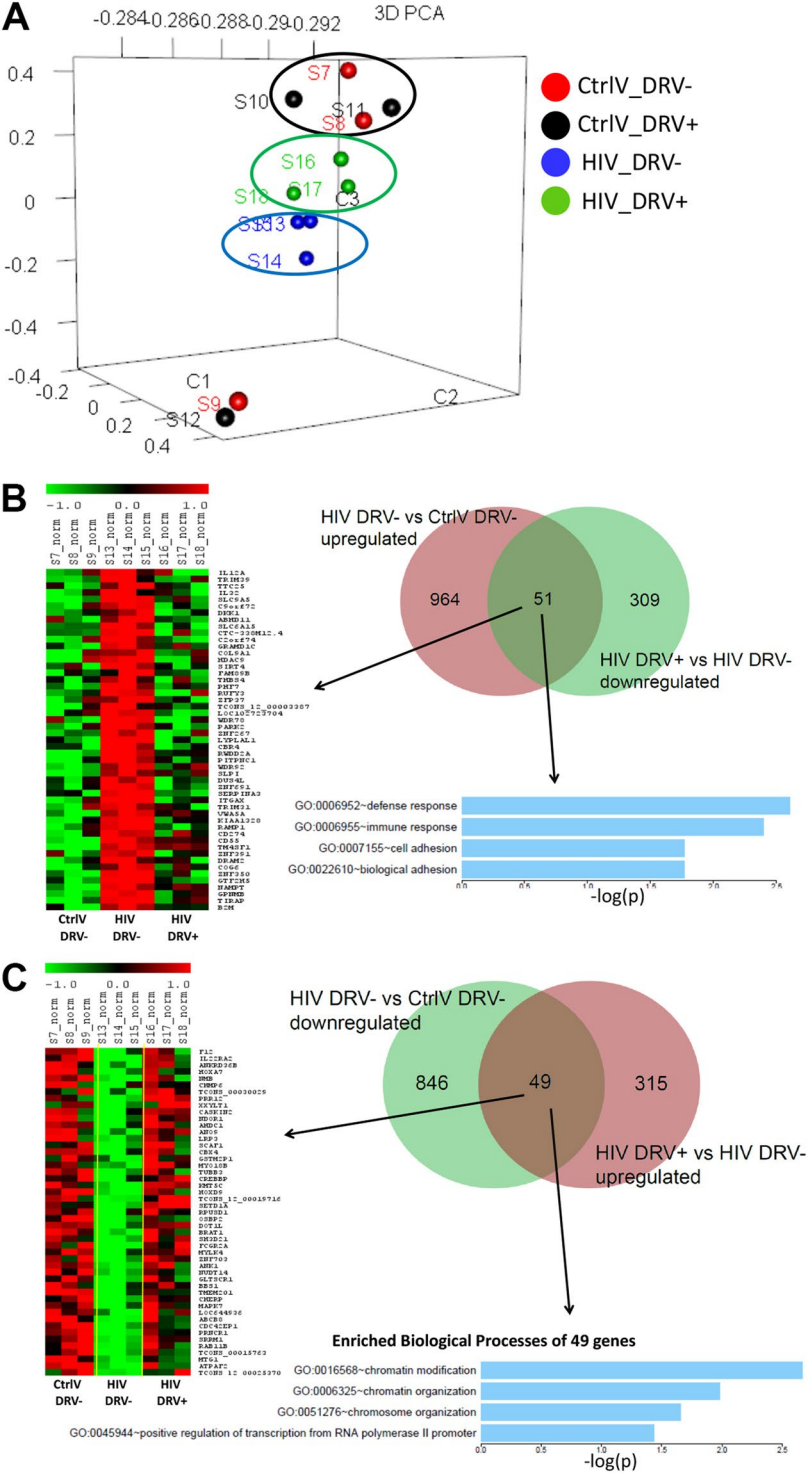
Principal component analysis demonstrated that EGFP control transduced cells clustered together regardless of DRV treatment. HIV-transduced cells clustered separately from EGFP-control cells and DRV treatment resulted in shift in gene expression of HIV-transduced cells closer to EFGP controls (**A**). HIV transduction increased expression of 1015 genes and DRV treatment of HIV-transduced cells decreased expression of 360 genes, 51 of which had been upregulated by HIV. Gene ontology pathway analysis revealed that these 51 genes had roles in immune defense and cellular adhesion (**B**). HIV transduction decreased expression of 895 genes and DRV treatment of HIV-transduced cells increased expression of 364 genes, 49 of which had been downregulated by HIV. Gene ontology pathway analysis revealed that these 49 genes had roles in chromatin modification and organization and transcription (**C**).

To determine the cellular pathways altered by DRV treatment independent of effects of HIV PR, we performed RNA-seq analysis of HIV (same *gag/pol*-deleted vector used in Fig. 1) and EGFP-control transduced HPT1b cells treated with DRV or vehicle control from day 4–7 post transduction. Principal component analysis demonstrated that EGFP-transduced cells clustered with or without DRV treatment. HIV-transduced cells clustered furthest from EGFP-transduced cells whereas DRV-treatment shifted HIV-transduced cells toward EGFP-transduced cells (Fig. 7A).

DRV treatment reduced expression of 51 of the 1,015 genes that were upregulated by HIV transduction. Pathway analysis of these 51 genes revealed that DRV downregulated genes involved in immune/defense responses and cell adhesion (Fig. 7B). Conversely, DRV increased expression of 49 of the 895 genes that were downregulated after HIV transduction and pathway analysis demonstrated that DRV restored expression of genes involved in chromatin/chromosomal modification and organization and RNA transcription (Fig. 7C). Since the HIV vector used in this study does not encode HIV PR, these effects were mediated via pathways that are independent of suppressing HIV PR.

## Discussion

Our data strongly suggest that the HIV PI DRV protects the kidneys from HIV-induced injury via mechanisms that are at least partly independent of suppression of HIV replication and expression of HIV PR. Previous research demonstrated that resolution of kidney disease in persons with HIVAN does not necessarily correlate with clearance of viral RNA from the kidney^15^, and the HIVAN phenotype in animal and *in vitro* models does not require viral replication and is recapitulated by expression of the HIV *vpr* and *nef* genes, which induce dysregulation of cellular processes including intracellular signaling, innate immune activation, cell death, and cell cycle^16,18–22^. Studies, mostly in the oncology literature, have reported effects of PIs on these same cellular pathways^44–46^.

Our data demonstrate that DRV prevents activation of several cellular signaling pathways in renal epithelial cells that had previously been found to be critical components of the pathogenesis of HIVAN, including Stat3, Erk, and Src. We also found that DRV reduced albuminuria, histologic kidney injury, and dysregulation of key molecular mediators of injury and inflammation in Tg26 mice. Further, DRV normalized expression of WT1 and synaptopodin in podocytes in Tg26 mice. Since the *in vitro* studies used HIV vectors lacking HIV PR and Tg26 mice develop the HIVAN phenotype in the absence of HIV PR or HIV replication, the protective effects of DRV in these studies could not have been mediated through suppression of HIV PR or viral replication. Moreover, our RNAseq data revealed that though DRV had minimal effects upon RNA expression in control-transduced cells, it prevented HIV-induced upregulation of immune response genes and downregulation of genes involved in chromatin modification/organization and RNA transcription. HIV-induced activation of inflammatory pathways is a key component of the pathogenesis of HIVAN^35,41,47^. Prior to availability of combination ART, several studies demonstrated that treatment with glucocorticoids was associated reduced progression to ESRD^48^ and the primary change in renal histopathology after treatment of HIVAN with prednisone was reduced tubulointerstitial inflammation^49^. Since studies have also implicated dysregulation of genes involved in chromatin maintenance and modification in HIV-induced kidney disease^50,51^, the ability of DRV to prevent HIV-induced down-regulation of genes involved in these pathways may be an important factor in its efficacy against HIV-induced injury of kidney cells.

The mechanism by which DRV exerted these pleiotropic effects is not clear but our data suggest that it is not mediated via inhibition of the 26S proteasome, as had previously been postulated in studies of HIV protease inhibitors^14,52^. Nonetheless, it remains possible that DRV has a subtle effect on proteasomal degradation of specific substrates that was not detected by our assays. Studies are underway in our laboratory to identify the cellular protein(s) that bind DRV and the data and pathway analyses from the RNAseq studies will be useful in narrowing the list of candidate proteins. Though DRV is a PI, it also inhibits non-protease enzymes including cytochrome P450 3 A^53^. It is therefore possible that the protective effects of DRV may not occur via inhibition of cellular protease(s).

In our *in vivo* studies, we found a significant protective effect of DRV against HIV-induced glomerular and tubular injury. Tg26 mice had severe albuminuria prior to the start of treatment and though DRV significantly reduced the severity of albuminuria, all Tg26 mice continued to have significant albuminuria throughout the duration of treatment. Despite continued albuminuria, glomerular and tubular histologic injury and molecular markers of HIV-induced glomerular and tubular injury were nearly completely ameliorated. These results suggest that either the severity of albuminuria dropped below a threshold required for renal injury during the 5 weeks of treatment or DRV protected the kidney from the deleterious effects of albuminuria, including activation of proinflammatory mediators.

HIV can infect parietal and visceral glomerular epithelial cells and RTEC^54^. In our *in vitro* studies, we focused upon the responses of RTEC rather than podocytes to HIV infection for several reasons. First, the severity of tubulointerstitial injury is the histopathologic parameter most strongly predictive of prognosis in most forms of kidney disease, including those characterized by severe proteinuria^55–57^. Second, RTEC comprise the great majority of renal parenchymal cells and is the renal compartment where most HIV infection occurs^25,54^. Third, previous studies demonstrated that the transcriptional response of our HPT1b RTEC line to transduction with the *gag/pol*-deleted HIV lentivirus used in these studies is nearly identical to primary RTEC infected with replication-competent HIV-1^35,47^. However, additional studies are needed to determine the molecular pathways that are affected by DRV in podocytes. Similarly, since our studies had previously demonstrated a critical role for Vpr-induced tubular injury in the pathogenesis of HIVAN^21,58^, we studied whether DRV prevents Vpr-induced injury in HPT1b cells. Though studies have implicated roles for Nef and Tat in podocyte injury, little is known regarding their role in RTEC injury.

There are several PIs approved for the treatment of HIV-1 and it is important to know whether our results are unique to DRV or are a “class effect” of PIs. Coppo *et al*. reported that saquinavir reduced need for immunosuppression in HIV-negative patients with steroid-resistant or -dependent nephrotic syndrome^14^ and several PIs, most notably nelfinavir, have been shown to have HIV-independent effects on cellular pathways in the oncology literature^44–46^. We chose to use DRV in these studies for two reasons. First, neither saquinavir or nelfinavir are commonly used due to the improved side effect profiles and dosing options of newer PIs such as DRV, which is now the most commonly prescribed PI. Second, DRV is less likely to cause kidney injury due to intratubular crystallization, which has been most commonly reported with indinavir^27,28^, but also can occur with atazanavir (especially ritonavir-boosted regimens), or lopinavir^29,30^. We believe that the effects of PI on the kidney may not unique to DRV. In addition to the published studies described above, we performed pilot studies with nelfinavir demonstrating similar effects as DRV upon cells *in vitro* and found in a small number of mice that daily intraperitoneal injection of nelfinavir for two weeks reduced renal expression of proinflammatory mediators in Tg26 kidneys (data not shown).

In our studies in Tg26 mice, AZT-treated mice statistically significant reduction in glomerulosclerosis and interstitial fibrosis and tubular atrophy but not cortical inflammation or tubular cast formation (Fig. 2). We did not find a reduction in albuminuria or changes in molecular markers of injury in AZT-treated mice. It is not clear whether AZT may have had a subtle protective effect that was independent of DRV or whether the histologic results reflected a false positive result. There was no evidence that treatment with DRV + AZT was superior to DRV alone.

Since there has not been a major resurgence in the incidence of HIVAN with the recent shift to treating many HIV-positive persons with INSTI-based (PI free) regimens, it is likely that suppression of viremia with subsequent improvement in immune function is also critical for prevention of HIVAN. However, it is plausible that in persons with existing proteinuric kidney disease, DRV might provide additional benefit compared to PI-free regimens. Our data, combined with the previous small case series suggesting a beneficial effect of saquinavir in proteinuric kidney disease in HIV-negative patients^14^, provide rationale for future studies to determine if DRV-containing regimens confer more renoprotection compared to INSTI-based regimens in PLWH with proteinuric CKD.

Novel strategies are urgently needed to prevent and treat renal disease in PLWH and in HIV-negative persons. This study suggests that DRV may confer protection against the deleterious effects of HIV gene expression via mechanisms that are independent of effects on PR or viral replication. Future studies should determine the optimal ART regimens for HIV-positive patients with kidney disease and to determine the potential role of ART medications in non-HIV related kidney diseases.

## Methods

### Cell culture and viral transduction

HPT1b is a human proximal tubular cell line that is conditionally immortalized with temperature-sensitive T antigen^33,34^. Cells were expanded at 33 °C and subsequently differentiated at 37 °C for two weeks prior to experiments. Cells were transduced with VSV-pseudotyped lentiviral vectors: NL4-3ΔG/P-EGFP (HIV), HR-VPR-IRES-EGFP (Vpr), HR-IRES-EGFP (EGFP control) and efficiency of transduction was assessed by EGFP fluorescence^21,58^. Lentiviral vectors were added to growth medium, incubated at 37 °C for 8 hours, then replaced with fresh medium. 72 hours later, DRV was added to the medium and cultured for 96 hours and then harvested for studies.

### Western blotting

HPT1b cells were lysed, the mixture was centrifuged and the supernatant was used for analysis. After electrophoresis, the protein samples were transferred to nitrocellulose membranes. The blots were subsequently incubated with rabbit polyclonal antibodies against p-Stat3, Stat3, p-Src, Src, p-Erk, Erk, p-JNK, JNK, p-P38 MAPK and P38 MAPK (Cell Signaling Technology Inc), followed by incubation with horseradish peroxidase-conjugated goat anti-rabbit IgG (Thermo Scientific) and detection by enhanced chemiluminescence (Thermo Scientific). Each experiment was repeated independently four times. For quantitative analyses, band densities were measured using ImageJ and ratios of p-Stat3/Stat3, p-Src/Src and p-Erk/Erk were calculated.

### Real-Time PCR

RNA was isolated using RNeasy Plus Mini Kits (Qiagen) and cDNA was made using SuperScript First-Strand Synthesis System (Invitrogen) and subjected to amplification using SYBR Green PCR Master Mix (Applied Biosystems) using an AB7500 cycler (Applied biosystems) according to the protocol described in supplementary methods. mRNA expression levels were quantified using the relative standard curve method according to the manufacturer’s instructions.

### Animal studies

Tg26 mice were assigned to 4 treatment groups (4 mice per group) and were treated with DRV (100 mg/kg), AZT (50 mg/kg), DRV + AZT, or vehicle control daily via oral gavage. Kidneys, urine, and blood were harvested after 5 weeks of treatment. Urinary ACR was quantified using a Mouse Albumin ELISA Quantification Set and Creatinine Colorimetric kit (Fisher Scientific). Animal studies were performed under protocols approved by the Institutional Animal Care and Use Committee at the Icahn School of Medicine at Mount Sinai and the Albert Einstein College of Medicine and all experiments were performed in accordance with relevant guidelines and regulations.

### Quantitative kidney histology scoring

Kidneys were perfused *in vivo* with 4 °C 4% paraformaldehyde in PBS and embedded in paraffin. Paraffin sections were cut at 2 microns and stained with periodic acid-Schiff (PAS) according to standard methods. For quantitative histologic analysis of PAS-stained kidneys, at least 100 glomeruli per mouse were evaluated by a renal pathologist (VD), who was blinded to treatment groups. Sections were scored for the % segmental and global glomerulosclerosis, % parenchyma with tubular casts and % cortex with tubular atrophy, interstitial fibrosis and interstitial inflammation.

### Immunofluorescence

Frozen kidney sections were permeabilized, blocked, and incubated with goat anti-Synaptopodin (Santa Cruz), Nephrin (R&D Systems), rabbit anti-WT1 (Abcam), Ki67 (Abcam), p-Stat3, p-Src, p-Erk, p-H2AX, or NF-κB p65 (Cell signaling), then incubated with Alexa 488- or 594-labeled donkey anti-rabbit IgG (Jackson Immunoresearch Laboratories) or fluorescein labeled Lotus Tetragonolobus Lectin (LTL) (VECTOR) and mounted in DAPI-Fluoromount-G Clear Mounting Media (SouthernBiotech). For antigen retrieval when staining for WT1, p-Stat3, p-Src, and p-H2AX, sections were heated in pH = 6 citrate buffer and permeabilized with Triton x-100 0.2% in phosphate buffered solution (PBS) for 5 minutes, followed by incubation with blocking solution of 5% BSA in PBS at room temperature for 30 minutes. Sections were imaged using an Axio Imager 2 and Observer microscopes (Zeiss). The ratio of cells expressing the protein of interest to total (DAPI + ) cells was calculated for each image. Fluorescence intensity for glomerular staining was measured using Image J and at least 160 glomeruli were evaluated in each group.

### Proteasome assays

HPT1b cells were transduced with no virus, HR-IRES-EGFP (EGFP control), NL4-3ΔG/P-EGFP (HIV), or HR-VPR-IRES-EGFP (Vpr) as above. Three days after transduction, the trypsin-like, chymotrypsin-like, and caspase-like proteasomal activities were assayed using the Proteasome Glo kit (Promega) according to manufacturer’s instructions. Luminescence was detected using a Varioskan Lux multimode plate reader (Thermo Scientific).

### Real-Time PCR

The following PCR cycler protocol and primers were used in quantitative real time PCR studies. PCR protocol: 95 °C 10 minutes, 40 cycles of 95 °C 15 seconds, 60 °C 60 seconds. Primers designed to detect murine IL-6 (sense: 5′-CAAAGCCAGAGTCCTTCAGAG, anti-sense: 5′-GCCACTCCTTCTGTGACTCC), human IL-6 (sense: 5′-AACCTGAACCTTCCAAAGATGG, anti-sense: 5′-TCTGGCTTGTTCCTCACTACT), human IL-8 (sense: 5′-TTTTGCCAAGGAGTGCTAAAGA, anti-sense: 5′-AACCCTCTGCACCCAGTTTTC), human IL-4 (sense: 5′- ACTTTGAACAGCCTCACAGAG, anti-sense: 5′- TTGGAGGCAGCAAAGATGTC), and human IL-7 (sense: 5′- TATTCCGTGCTGCTCGCAAGTTGA, anti-sense: 5′- ACTCTTTGTTGGTTGGGCTTCACC). Cyclophilin A was used as a housekeeping control (murine cyclophilin: sense: 5 ′-AGGGTGGTGACTTTACACGC, antisense: 5′-ATCCAGCCATTCAGTCTTGG; human cyclophilin: sense: 5 ′-CAGACAAGGTCCCAAAGACAG, antisense: 5′-TTGCCATCCAACCACTCAGTC).

### RNA-sequencing and data analysis

Sample quality control analysis, cDNA library synthesis, and RNA sequencing were performed by the Weill Cornell Genomics Core. Briefly, RNA quality was confirmed using an Agilent bioanalyzer (Agilent). cDNA libraries were synthesized using TruSeq RNA Sample Preparation (Illumina) and libraries were sequenced on a HiSeq. 4000 sequencer (Illumina) with single-end 51 cycles. Analysis of RNAseq data was performed as previously^50^. The differential analysis by paired LIMMA was performed to identify genes that were significantly dysregulated in HPT1b cells after HIV transduction and/or DRV treatment at adjusted FDR p value < 0.05. Differentially expressed genes were then subjected to gene ontology function and canonical pathway enrichment analysis by Fisher exact test. RNAseq data were submitted to the National Center for Biotechnology Information Gene Expression Omnibus Repository (accession #GSE136340).

### Statistical analyses

With the exception of RNAseq data analysis, statistical comparisons between groups were performed using the ANOVA single factor analysis tool in Excel (Microsoft). Statistical significance was defined as two-sided p < 0.05.

## Supplementary information

Supplementary figures

## Data Availability

The datasets generated during and/or analyzed during the current study are available from the corresponding author on reasonable request. RNAseq data are available from National Center for Biotechnology Information Gene Expression Omnibus Repository (accession #GSE136340).
