# Publisher Correction: The HIV protease inhibitor darunavir prevents kidney injury via HIV-independent mechanisms

**DOI:** 10.1038/s41598-020-60085-4

**Published:** 2020-03-04

**Authors:** Xiaobo Gao, Alan Rosales, Heidi Karttunen, Geetha M. Bommana, Buadi Tandoh, Zhengzi Yi, Zainab Habib, Vivette D’Agati, Weijia Zhang, Michael J. Ross

**Affiliations:** 1 Division of Nephrology, Albert Einstein College of Medicine/Montefiore Medical Center, Bronx, NY USA; 2 BronxCare Health System, Bronx, NY USA; 3 Division of Nephrology, Icahn School of Medicine at Mount Sinai, New York, NY USA; 4 New York University, New York, NY USA; 5 Department of Pathology, Columbia University, College of Physicians & Surgeons, New York, NY USA; 6 Department of Development and Molecular Biology, Albert Einstein College of Medicine, Bronx, NY USA

Correction to: *Scientific Reports* 10.1038/s41598-019-52278-3, published online 01 November 2019

The original version of this Article contained a typographical error in the spelling of the author Vivette D’Agati, which was incorrectly given as Vivette D’ Agati. This has now been corrected in the PDF and HTML versions of the Article.

