# Supplementary material for: The HIV protease inhibitor darunavir prevents kidney injury via HIV-independent mechanisms
Source: Sci Rep. 2019 Nov 1;9:15857. doi: 10.1038/s41598-019-52278-3 (PMC6825220; doi:10.1038/s41598-019-52278-3)

Supplementary Figures

The HIV protease inhibitor darunavir prevents kidney injury via HIV-independent mechanisms

Xiaobo Gao[1], Alan Rosales[1], Heidi Karttunen[1], Geetha M. Bommana[2], Buadi Tandoh[1], Zhengzi Yi[3], Zainab Habib[4], Vivette D'Agati[5], Weijia Zhang[3], and Michael Ross[1,6]

[1]Division of Nephrology, Albert Einstein College of Medicine/Montefiore Medical Center, Bronx, NY, USA; [2]BronxCare Health System, Bronx, NY, USA; [3]Division of Nephrology, Icahn School of Medicine at Mount Sinai, New York, NY, USA; [4]New York University, New York, NY, USA; [5]Department of Pathology, Columbia University, College of Physicians & Surgeons, New York, NY, USA; [6]Department of Development and Molecular Biology, Albert Einstein College of Medicine, Bronx, NY, USA

Figure S1

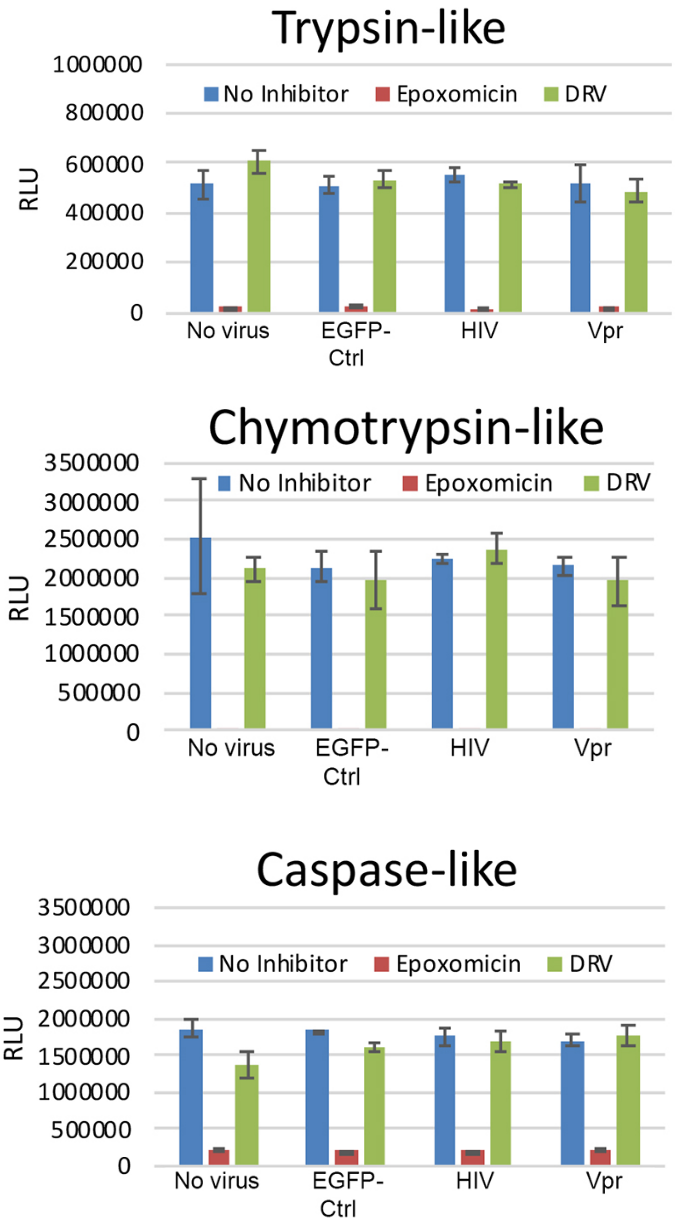

Figure S1. Darunavir did not significantly reduce 26S proteasome activity in HPT1b cells. Assays using luminescent substrates specific for the caspase-, chymotrypsin-, and trypsin-like proteasome activities did not demonstrate inhibition after treatment with DRV in HIV- or Vpr-transduced cells. Epoxomicin blocked all proteasomal activities. RLU= relative luminescence units. Error bars +/- SD.

Uncropped western blot images used in Figure 1a
Erk and p-Erk

Western Blot-2015-12-15

Erk1/2 MW: 42/44 kD

HPT1b cell in 6-well plate
100% confluent cells

1: Control-No virus
2: Control-No Virus-DRV
3: HR-EGFP
4: HR-EGFP-DRV-5d
5: NL4-3(1:12.5 virus dilution)
6: NL4-3(1:12.5)-DRV-5d
7: NL4-3(1:6.25 virus dilution)
8: NL4-3(1:6.25)-DRV-5d
9: Vpr (viral prep #1)
10: Vpr (viral prep #1)-DRV-5d
11: Vpr (viral prep #2)
12: Vpr (viral prep #2)-DRV-5d

Erk 1/2
Rabbit mAb

Phospho-Erk1/2
Rabbit mAb

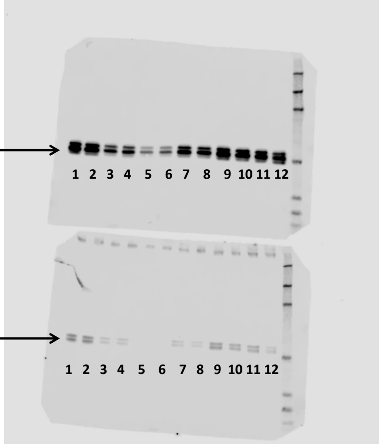

Western Blot-2015-12-17

Erk1/2 MW: 42/44 kD

HPT1b cell in 6-well plate
100% confluent cells

1: NL4-3(1:12.5 virus dilution)
2: NL4-3(1:12.5)-DRV-5 days
3: Control-No virus
4: Control-No Virus-DRV
5: HR-EGFP
6: HR-EGFP-DRV-5 days
7: Vpr (viral prep #1)
8: Vpr(viral prep #1)-DRV-5d
9: Vpr(viral prep #2)
10: Vpr(viral prep #2)-DRV-5d

Erk1/2
Rabbit mAb

Phospho-Erk1/2
Rabbit mAb

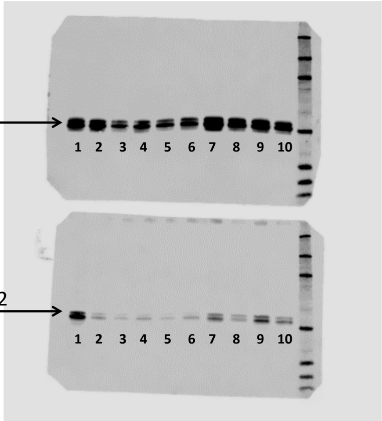

Western Blot-2015-12-23

Erk1/2 MW: 42/44 kD

HPT1b cell in 6-well plate
100% confluent cells

1: Control-No virus
2: Control-No Virus-DRV
3: HR-EGFP
4: HR-EGFP-DRV-5d
5: NL4-3(1:12.5 virus dilution)
6: NL4-3(1:12.5)-DRV-5d
7: NL4-3(1:6.25 virus dilution)
8: NL4-3(1:6.25)-DRV-5d
9: Vpr (viral prep #1)
10: Vpr (viral prep #1)-DRV-5d
11: Vpr (viral prep #2)
12: Vpr (viral prep #2)-DRV-5d

Erk1/2
Rabbit mAb

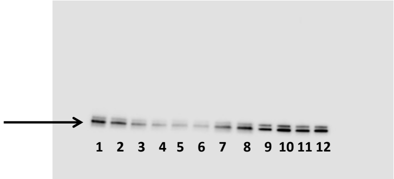

Phospho-Erk1/2
Rabbit mAb

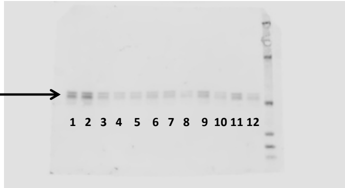

Uncropped western blot images used in Figure 1a
Src and p-Src

Western Blot-2016-03-10

Src MW: 60 kD

HPT1b cell in 6-well plate
100% confluent

1: HR-EGFP
2: HR-EGFP-DRV-5d
3: NL4-3(1:12.5 virus dilution)
4: NL4-3(1:12.5)-DRV-5d
5: NL4-3(1:6.25)
6: NL4-3(1:6.25)-DRV-5d
7: Vpr(virus prep #1)
8: Vpr(virus prep #1)-DRV-5d
9: Vpr(virus prep #2)
10: Vpr(virus prep #2)-DRV-5d

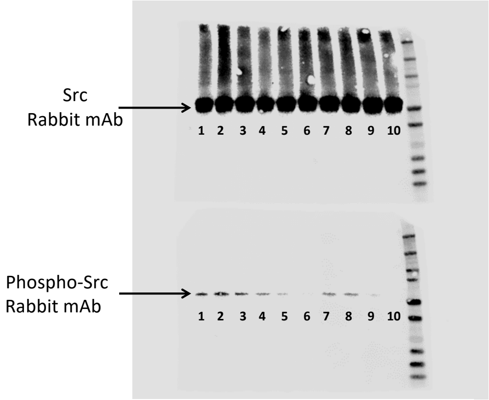

Western Blot-2016-04-06

Src MW: 60 kD

HPT1b cell in 6-well plate
100% confluent

1: HR-EGFP
2: HR—EGFP-DRV-5d
3: NL4-3(1:12.5 virus dilution)
4: NL4-3(1:12.5)-DRV-5d
5: NL4-3(1:6.25)
6: NL4-3(1:6.25)-DRV-5d
7: Vpr (virus prep #2)
8: Vpr (virus prep #2)-DRV-5d

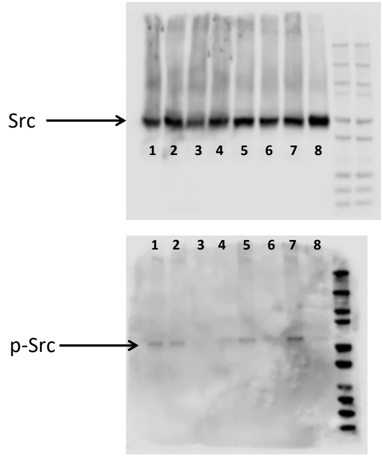

Western Blot-2016-05-05

Src MW: 60 kD

HPT1b cell in 6-well plate
100% confluent

1: NL4-3(1:12.5 virus dilution)-DRV-5d
2: NL4-3(1:12.5)
3: HR-EGFP
4: HR-DRV-5d
5: Vpr (virus prep #2)
6: Vpr (virus prep #2)-DRV-5d

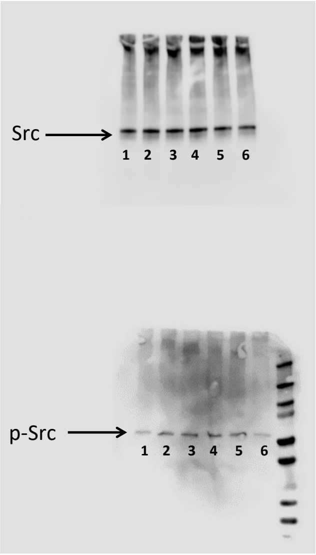

Uncropped western blot images used in Figure 1a
Stat3 and p-Stat3

Western Blot-2015-12-17

Stat3 MW: 79/86 kD

HPT1b cell in 6-well plate
100% confluent cells

1: HR-EGFP
2: HR-EGFP-DRV-5d
3: NL4-3(1:12.5 virus dilution)
4: NL4-3(1:12.5)-DRV-5d
5: NL4-3(1:6.25)
6: NL4-3(1:6.25)-DRV-5d
7: Vpr (virus prep #1)
8: Vpr (virus prep #1)-DRV-5d
9: Vpr (virus prep #2)
10: Vpr (virus prep #2)-DRV-5d

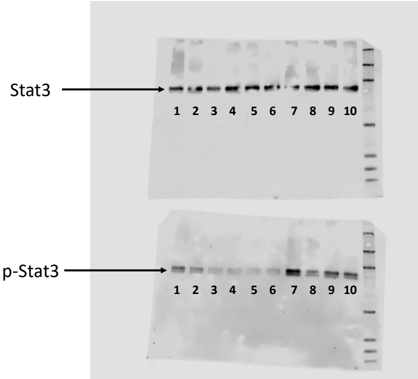

Western Blot-2016-01-05

Stat3 MW: 79/86 kD

HPT1b cell in 6-well plate
100% confluent cells

1: Control-No virus
2: Control-No virus-DRV
3: HR-EGFP
4: HR-EGFP-DRV-5d
5: NL4-3(1:12.5 virus dilution)
6: NL4-3(1:12.5)-DRV-5d
7: NL4-3(1:6.25)
8: NL4-3(1:6.25)-DRV-5d
9: Vpr (virus prep #1)
10: Vpr (virus prep #1)-DRV-5d
11: Vpr (virus prep #2)
12: Vpr (virus prep #2)-DRV-5d

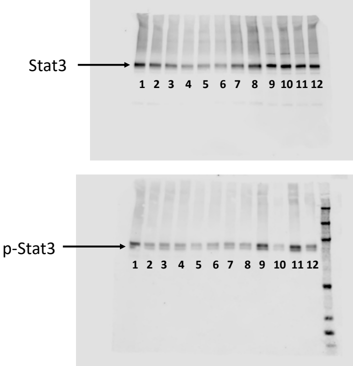

Western Blot-2016-05-24

Stat3 MW: 79/86 kD

HPT1b cell in 6-well plate
100% confluent cells

1: HR-EGFP
2: HR-EGFP-DRV-5d
3: NL4-3(1:12.5 virus dilution)
4: NL4-3(1:12.5)-DRV-5d
5: NL4-3(1:6.25)
6: NL4-3(1:6.25)-DRV-5d
7: Vpr (virus prep #1)
8: Vpr (virus prep #1)-DRV-5d
9: Vpr (virus prep #2)
10: Vpr (virus prep #2 DRV-5d

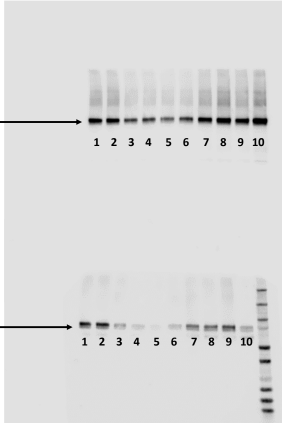

Western Blot-2018-09-27

Stat3 MW: 79/86 kD
Membranes cut prior to blotting

HPT1b cell in 6-well plate
100% confluent cells

1: HR-EGFP
2: HR-EGFP-DRV-5d
3: NL4-3(1:12.5 virus dilution)
4: NL4-3(1:12.5)-DRV-5d
5: NL4-3(1:6.25)
6: NL4-3(1:6.25)-DRV-5d
7: Vpr (virus prep #1)
8: Vpr (virus prep #1)-DRV-5d
9: Vpr (virus prep #2)
10: Vpr (virus prep #2)-DRV-5d

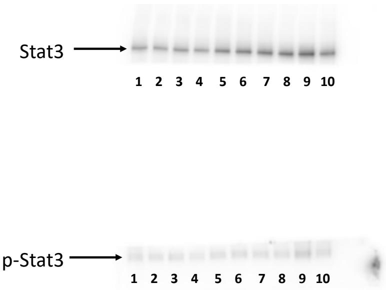

Uncropped western blot images used in Figure 1a
JNK, p-JNK

Western Blot-2019-08-28

JNK MW: 47 kD

HPT1b cell in 6-well plate
100% confluent cells

1: HR-EGFP
2: HR-EGFP-DRV-5d
3: NL4-3 prep#1 (1:12.5 dilution)
4: NL4-3 prep#1 (1:12.5 dilution)-DRV-5d
5: NL4-3 prep#2 (1:12.5 dilution)
6: NL4-3 prep#2 (1:12.5 dilution)-DRV-5d
7: NL4-3 (1:6.25 dilution)
8: NL4-3(1:6.25 dilution)-DRV-5d
9: Vpr (prep#1)
10: Vpr(prep#1)-DRV-5d
11: Vpr(prep#2)
12: Vpr(prep#2)-DRV-5d

p-JNK

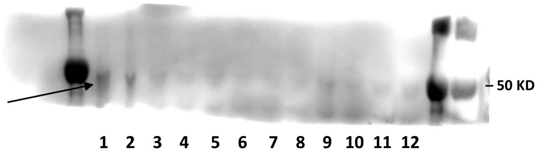

total JNK

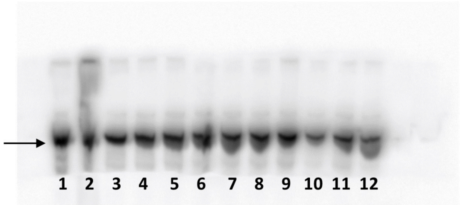

Uncropped western blot images used in Figure 1a
p38, p-p38

Western Blot-2019-08-30

p38 MW: 40 kD

HPT1b cell in 6-well plate
100% confluent cells

1: HR-EGFP
2: HR-EGFP-DRV-5d
3: NL4-3 prep#1 (1:12.5 dilution)
4: NL4-3 prep#1 (1:12.5 dilution)-DRV-5d
5: NL4-3 prep#2 (1:12.5 dilution)
6: NL4-3 prep#2 (1:12.5 dilution)-DRV-5d
7: NL4-3 (1:6.25 dilution)
8: NL4-3(1:6.25 dilution)-DRV-5d
9: Vpr (prep#1)
10: Vpr(prep#1)-DRV-5d
11: Vpr(prep#2)
12: Vpr(prep#2)-DRV-5d

p-p38

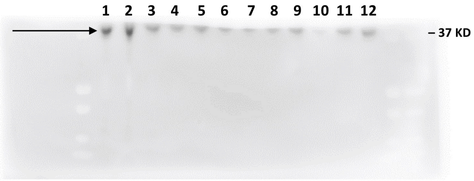

p-p38

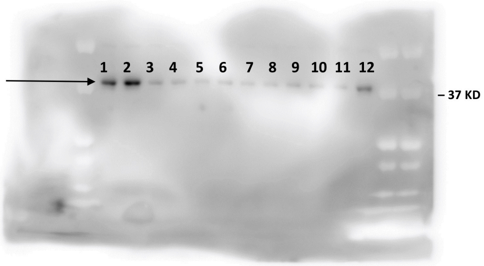

total p38

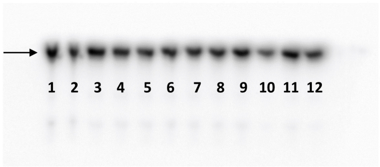

Supplement: Supplementary file 1 — Supplementary figures [file 41598_2019_52278_MOESM1_ESM.pdf]
